# The Unification of the Medical Profession Again

**Published:** 1902-06

**Authors:** 


**A Monthly Review of Medicine and Surgery.**

*EDITOR:*

WILLIAM WARREN POTTER, M. D.

All communications, whether of a literary or business nature, books for review and exchanges should be addressed to the editor: 284 FRANKLIN STREET, BUFFALO, N. Y.

VOL. XLI.—LVII. JUNE, 1902. NO. 11.

### The Unification of the Medical Profession Again.

LAST month we directed the attention of our readers to a singularly interesting utterance upon the subject of the "Coming unification of the medical profession." In this issue we are permitted to notice facts of a most impressive character, showing the rapid progress of events, facts which permit us to claim without likelihood of successful controversy that, in this country, the virtual unification of the medical profession is an established fact.

In support of this view the fourth volume of Wood's Reference Handbook of the Medical Sciences, just issued, contains an article by James Russell Parsons, Jr., of Albany, secretary of the University of the State of New York, entitled, Examining and licensing boards, giving in a lucid and modest way most significant facts as to the rise and progress of state licensure, and as to the coincident progress in improvement of the management of our medical schools.

We invite attention to this article and proceed to apply some of the facts therein noted to the present medical situation. The preliminary work of unification of the medical profession of this state was inaugurated by the Medical Society of the State of New York, under the presidency of the eminent Dr. Abraham Jacobi, in the year 1881, through a movement which finally effected the repeal of the code of ethics, an instrument of many virtues, but in some of its provisions openly antagonistic to the medical laws of the state.

It is interesting to recall that at this time, 1881, there were in this country 5 schools of a three years' course, and 103 schools of a two years' course, while there was not one school of a four years' course. In the year 1899 we find 141 schools with a four years' course, 10 schools with a three years' course and 2 schools with a two years' course. Examination of the various faculties reveals similar growth as to the number of teachers and the length of term. Then, two years, seven teachers and three months was the rule; now, four years, scores of teachers, and six months is the mode,—and from then to now, that is from 1881 to 1899, is only eighteen years!

This is certainly remarkable progress in medical improvement, and that the physicians having the superior advantages in professional training and preparation would, from their increased knowledge, incline their hearts to medical unity was the hope and the legitimate expectation of the men active in directing the movement. How that hope and expectation has met fruition, —the extent of the growth in medical unity, the close and rapid following of medical unity upon improved standards of medical education, of increased efficiency in preparedness for the practice of the healing art,—the article above mentioned points out and the view is glorious.

Medical unity in theory, in fact, and in action, invoking the majesty of the law to demand state examination and state licensure to practise medicine, is seen to have spread from its inception in this state and is now in efficient operation in fifty-one of the political divisions of the country; and the mixed examining and licensing boards, the boards composed of physicians, homeopaths and eclectics, meeting and consulting in practical equality, fraternity and harmony,—the precise medical situation the code of ethics was depended upon to prevent,—are now protecting the medical honor, and the public health from the incompetent medical graduates, in Alabama, Arizona, Arkansas, California, Colorado, Hawaii, Idaho, Illinois, Indian Territory, Indiana, Iowa, Kansas, Kentucky, Maine, Massachusetts, Michigan, Minnesota, Mississippi, Missouri, Montana, Nebraska, Nevada, New Jersey, New Mexico, North Carolina, North Dakota, Ohio, Oklahoma, Oregon, Rhode Island, South Carolina, South Dakota, Tennessee, Utah, Virginia, Washington, West Virginia, Wisconsin, Wyoming.

Important, significant and satisfactory as are these milestones of the march of medical improvement, progress and unity, there are still others, and at least one other should be mentioned.

For nearly half a century, or since 1857, there has been on the part of the medical profession in almost every state in the union mutiny and rebellion, with organisation if not by force of arms, against the will of the people, as expressed in the laws of the land. Good men have been wont to retort, when it was pointed out that certain medical practice, custom or tradition, was contrary to the law and the constitution, "So much the worse for the constitution," and have thought that thereby they exhibited laudable devotion to the higher interests of ethics and medical science; whereas they now see that obedience to the law is the highest ethics, and that progress under the law and the constitution is not only both feasible and positive, but expedient.

The reorganisation of the American Medical Association with a constitution broad, catholic and simple, by practical unanimity, whereby the medical profession of this country is asked to unite for noble purposes, is a sign of the times, a bow of promise, a certain indication that through the narrow gate of obedience to the law, the medical profession has entered into such intimate and sympathetic relations with the people as to make the future radiant with hope. In this cause the medical profession of the Empire State through its executive organisation, the Medical Society of the State of New York, has borne a part at once initiative, conspicuous, and honorable.

